# Major Molecular Signaling Pathways in Oral Cancer Associated With Therapeutic Resistance

**DOI:** 10.3389/froh.2020.603160

**Published:** 2021-01-25

**Authors:** Saima Usman, Ahmad Jamal, Muy-Teck Teh, Ahmad Waseem

**Affiliations:** Centre for Oral Immunobiology and Regenerative Medicine, Institute of Dentistry, Barts and The London School of Medicine and Dentistry, Queen Mary University of London, London, United Kingdom

**Keywords:** signaling pathways, therapeutic resistance, genetic lesions, head & neck cancers, oncogenic mutations

## Abstract

Oral cancer is a sub-category of head and neck cancers that primarily initiates in the oral cavity. The primary treatment option for oral cancer remains surgery but it is associated with massive disfigurement, inability to carry out normal oral functions, psycho-social stress and exhaustive rehabilitation. Other treatment options such as chemotherapy and radiotherapy have their own limitations in terms of toxicity, intolerance and therapeutic resistance. Immunological treatments to enhance the body's ability to recognize cancer tissue as a foreign entity are also being used but they are new and underdeveloped. Although substantial progress has been made in the treatment of oral cancer, its complex heterogeneous nature still needs to be explored, to elucidate the molecular basis for developing resistance to therapeutic agents and how to overcome it, with the aim of improving the chances of patients' survival and their quality of life. This review provides an overview of up-to-date information on the complex role of the major molecules and associated signaling, epigenetic changes, DNA damage repair systems, cancer stem cells and micro RNAs in the development of therapeutic resistance and treatment failure in oral cancer. We have also summarized the current strategies being developed to overcome these therapeutic challenges. This review will help not only researchers but also oral oncologists in the management of the disease and in developing new therapeutic modalities.

## Introduction

Oral cancer is a subcategory of head and neck cancers that initiates inside the mouth involving anterior two-thirds of the tongue, gingivae, mucosal lining of lips and cheeks, sublingual floor of the mouth, the hard palate and the small retromolar area [1, 2]. Signs and symptoms associated with oral cancer include a lump or non-healing sore/ulcer present for more than 14 days, presence of soft red, white or speckled (red and white) patches in the mouth, difficulty in swallowing, chewing, speaking, jaw or tongue movements, malocclusion or ill-fitting dentures and sudden weight loss [3].

Oral cancers are the 6th leading cancer by incidence in the world and 90% of these are histologically squamous cell carcinoma [4]. The 5-year survival rate is <50% in advanced cases with women having a more favorable outcome [5]. The prognosis of these patients is always reliant on age, lymph node involvement and primary tumor size and location [6]. The most common risk factors include the premalignant conditions, consumption of tobacco, betel nut, alcohol along with poor oral hygiene, UV radiations, Epstein Barr Virus (EBV) and Human Papilloma Virus (HPV) especially HPV 16 and 18 [7].

The extent of oral cancer spread is estimated by staging the cancer. The commonly used staging system for oral cancer is TNM system, where T (for tumor) defines the size of the primary tumor. It is further categorized from 1 to 4 on the basis of tumor size, a higher number indicates larger size. N (for lymph nodes) shows extend of cancer spread to lymph nodes in the vicinity of the organ. It is further categorized to N_0_ (no spread), N_1_, N_2_, or N_3_. The N_1_-N_3_ shows the number of lymph nodes involved alongside their location and size. M (for metastasis) describes cancer spread to other parts of the body via lymph or blood. It is further classified to M_0_ (no spread) and M_1_ (spread). Overall oral cancer staging is given as follows [8]:

Stage 0 – Carcinoma *in situ*,
Stage 1 – Smaller tumor that has not grown out of the organ in which it began
Stages 2 and 3 –Larger tumor that has grown outside the organ, in which it began, to the nearby tissues.
Stage 4 – Spread of cancer to distant areas of the body via blood or lymphatic system (metastatic spread).

### Early Diagnosis Is the Key to Enhancing Patient Survival

Oral cancer is diagnosed on the basis of routine visual physical examination, medical history and risk factors probing. Early-stage diagnosis and prompt referral to specialist hospitals is a crucial factor in increasing the patients' survival rate of up to 90%. Unfortunately, about 60% of oral cancers are diagnosed at advanced stages III or IV with metastasis leading to a higher mortality rate [9]. Diagnostic delays may be attributed to both patients' ignorance as well as the ignorance of health care professionals [10]. The delaying factors on the patients' behalf include late perception of the lesion or symptoms as oral cancers are mostly asymptomatic, ignoring the lesions, self-medications, fear of surgery, poor socio-economic conditions and little or no access to specialized healthcare [11]. On the professional side, the factors include improper intra-oral and extra-oral examination, delay in biopsy taking, or wrongful biopsy site selection for histopathological examination. The average delay for the initial to definite diagnosis is reported to be about 6 months [12].

Population targeted educational intervention should mainly focus on the high-risk groups. The professional educational interventions, on the other hand, should include a sound knowledge of the disease presentation, specifically on sites like gingivae, floor of the mouth, and retromolar trigone. Screening programs should also be implemented in every country at primary or the secondary care level. The use of social media and mobile apps can be beneficial for population targeted oral cancer symptoms awareness programs [13].

### Treatment Options

The stage of the disease usually determines the primary option for the treatment of oral cancer. The treatment options include surgical resection, chemotherapy, radiotherapy, immunotherapy alone or in combination [14]. Despite favorable advancements in the conventional therapeutic modalities, many disadvantages still need to be addressed; surgical resection may lead to long-lasting disfigurements, multiple corrective surgeries usually cause considerable deformities that leaves patients in psycho-social stress and isolation, whereas radio- or chemo- therapies end up with significant toxicities or treatment resistance, all compromising the patients' quality of life and well-being [15]. Also, locoregional relapse may occur after years of the treatment leading to recurrent growth of the cancers [16].

**Figure 1:**
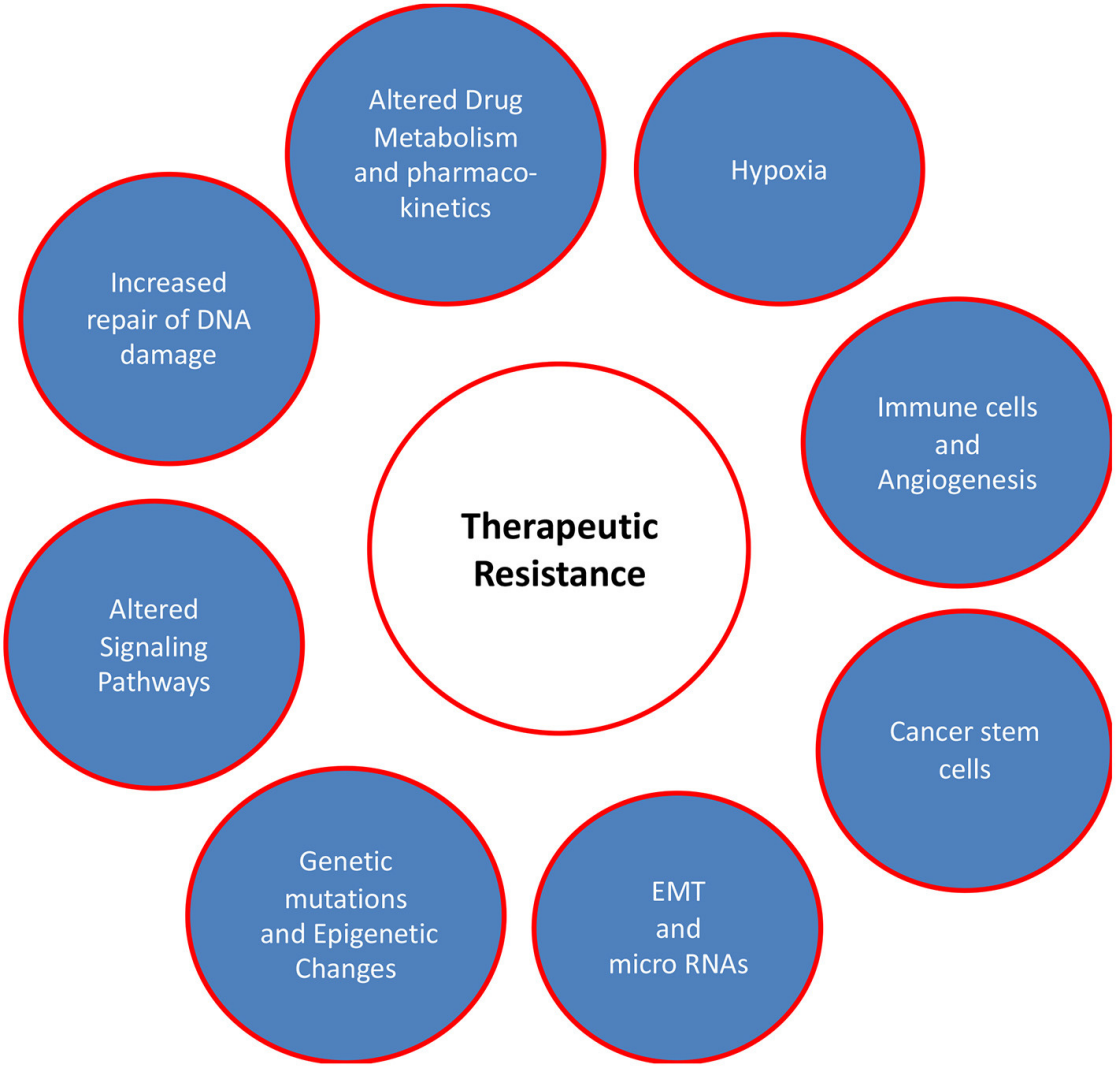
Factors responsible for therapeutic resistance in oral cancer patients. This review focusses on the complex multiple factors including genetic alterations, dysregulated signaling pathways, EMT/micro RNA and hyperactive DNA damage repair systems. Other factors such as microenvironment (hypoxia), drug metabolism/pharmacokinetics, and immune cells/angiogenesis are also critical in conferring therapeutic resistance in oral cancer, but they have been excluded from this review to maintain the focus.

The effectiveness of different therapeutic modalities is largely dependent on the mutational profile of tumors as genetic alterations confer new oncogenic potential to cancer cells. The precise targeting of these alterations together with treatment regimen modifications decreases therapeutic resistance and may result in countless lives being saved from potential morbidity and mortality. The factors responsible for therapeutic resistance are discussed here and are summarized in Figure 1.

## Genetic Factors and Signaling Pathways Involved in Therapeutic Resistance of Oral Cancer

### TP53

*TP53* is a tumor suppressor gene which prevents carcinogenesis by instigating G1 cell cycle arrest. Activated p53 (protein product of *TP53*) is a DNA-binding transcription factor that targets different proteins that are either involved in apoptosis (e.g., Bad, Bax, Puma, Fas, Apaf1, Noxa) or can induce cell cycle arrest (e.g., BTG2, CDNK1/p21/pRb/E2F1 pathway, GADD45) and activate DNA repair mechanisms (e.g., p48, XPC, PCNA, DDB2) after exposure to UV light, ionizing radiation or other DNA-damaging agents [17]. Because radiation therapy and chemotherapeutic agents act through many of these common pathways requiring the same proteins, p53 also plays a central role in the effective response to these cancer therapies [18].

*TP53* gene is reported to encode at least 15 isoforms of p53 arising through different transcription initiation sites and alternative splicing [19]. Due to this isoformic nature, the molecular mechanisms behind the role of p53 in cancer progression and therapeutic resistance are very complex. Its categorization as a single class of tumor suppressor gene is therefore not possible. Mutant p53 cannot be considered as a single entity, but a multifaceted collection of proteins, each with a unique collection of properties such as dominant-negative functionality (antimorphic), heterogeneous loss of activity, and functional gain (neomorphic) [20]. About 40–70% of oral cancers have mutations in the *TP53* gene, leading to non-functioning product. More than 90% of these mutations are between exons 5 and 8 of *TP53*, a region where most common mutations include R175, G245, R248, R249, R273, and R282 on the DNA binding domain [21, 22]. *TP53* mutations can be segregated into disruptive or non-disruptive categories [18]. Disruptive mutations comprise irregularities in the DNA binding domains or a truncated p53 due to the existence of an early stop codon; these variations cause substantial loss of function [23]. As a result, apoptosis or cell-cycle arrest is restricted leading to tumor cell survival and treatment failures. In contrast, non-disruptive mutations partly affect the normal functionality of p53. Inside cancer cells with non-disruptive mutant p53, repair of damaged DNA is insufficient however functional enough to produce heterogeneous clones of tumor cells with novel oncogenic features and therefore known as gain-of-function mutations (neomorphic). As a result, DNA damage-induced cell death via downregulation of pro-apoptotic genes and upregulation of pro-survival genes is minimized. Such gain of function mutations can also increase DNA repair, genomic instability, stimulate proliferation, invasion, migration and dysregulate metabolism, collectively leading to therapeutic resistance [24]. Disruptive and non-disruptive mutations are linked with resistance to standard anticancer drugs such as Cisplatin, EGFR-inhibitors, alkylating agents, antioestrogens, anthracyclines, and antimetabolites [21]. The mechanism of conferring drug resistance by mutant p53 in each of these drug groups is not identical and is beyond the scope of this article.

Apart from somatic and germline mutations, wild-type p53 functions can be disrupted by alterations in its regulatory pathways. Thus, understanding the mechanism(s) behind the inactivation of p53 is very important for personalized treatment strategies. The p53 levels in normal cells are well regulated. Usually, Mouse Double Minute 2 (MDM2) negatively regulates p53 by binding to its trans-activating domain (TAD) and ubiquitinylates so that it can be degraded. p53 also stimulates transcription of MDM2, hence they are balanced via negative feedback mechanism. Stress conditions such as DNA damages can alter this balance toward increased p53 levels [25]. Based on this tightly balanced negative feedback mechanism; it is understandable that any genetic polymorphism in MDM2 can also alter p53 functional levels. A number of reports have highlighted this fact in oral cancers especially via one intronic polymorphism (rs2279744) in MDM2 leading to its induced expression [26].

#### HPV Infections and Their Association With p53 Protein

High-risk HPV (hr-HPV) types 16 and 18 have oncogenic potential [27]. Several reports have proposed that p53 mutations along with HPV infections have higher recurrence and poor prognosis in oral cancer patients [28]. The oncoprotein E6 of HPV degrades p53, and therefore cells carrying damaged DNA enter irrationally into mitosis, keep proliferative capacity and collectively leading to chromosomal defects in hr-HPV associated cancers [29]. The hr-HPV positive oral cancers without p53 mutations have overall good prognosis and are treatment sensitive as compared to HPV negative. One possible reason for good prognosis may be the efficient role of wild-type p53 that has escaped hr-HPV mediated degradation and has triggered cellular apoptosis during cancer therapy [30].

#### Treatment Strategies Targeting p53

**Figure 2:**
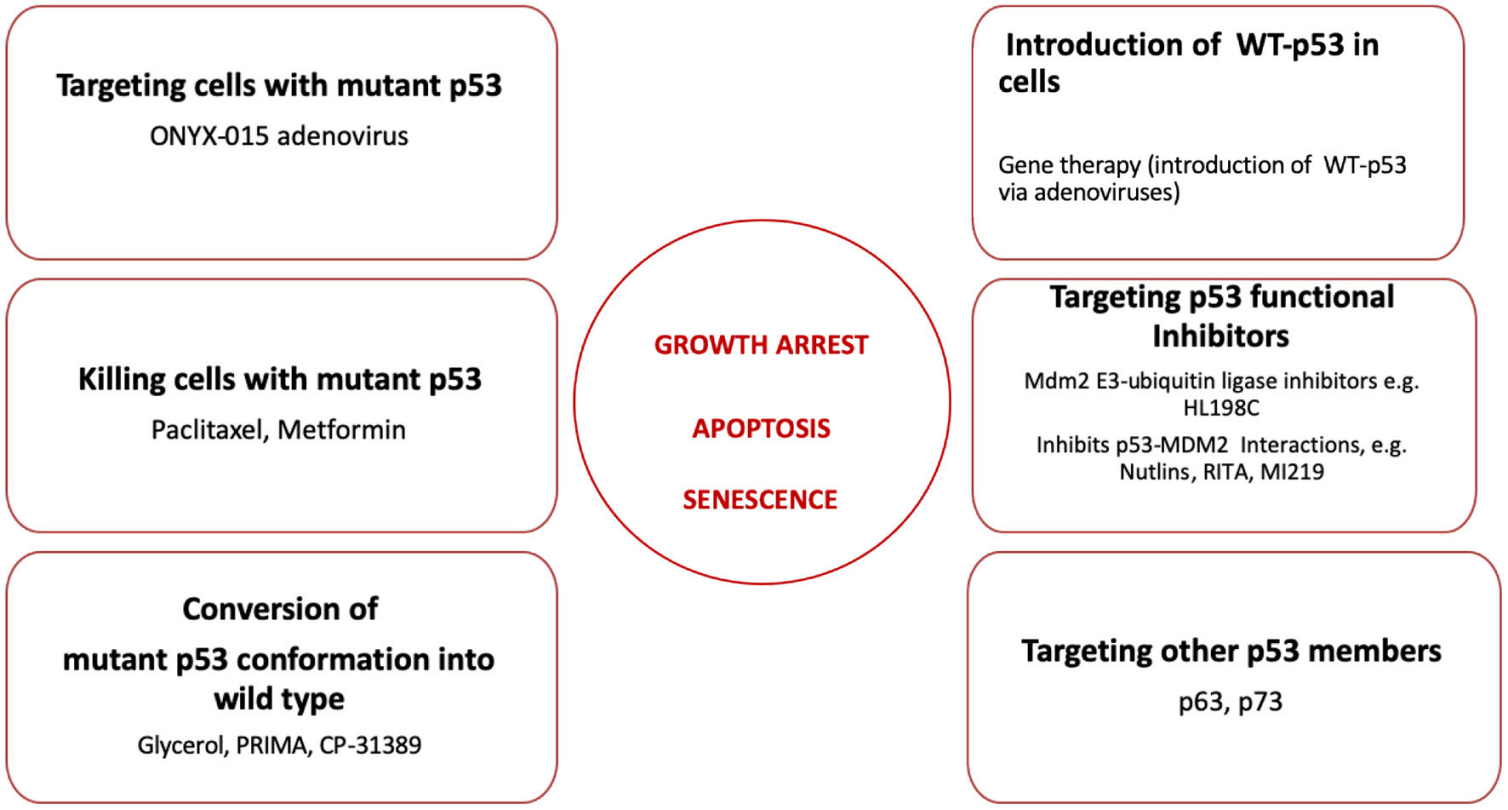
Strategies to restore p53 functions including growth arrest, induction of apoptosis and senescence during oral cancer treatment. Six different strategies to restore p53 functions discussed in this review are listed here. The proposed reagents employed in every strategy are summarized and discussed in the text.

A number of different approaches have been developed to restore the function of mutant p53 in oral cancers. These are summarized in Figure 2.

##### Gene Therapy

Gene therapy is intended to insert genetic material into cancer cells to recompense abnormal genes or to make a beneficial protein. Viruses are genetically engineered into vectors to deliver a gene but do not cause any disease by themselves [31]. The commonly employed vector in p53 gene therapy in oral cancers is the adenovirus due to its extraordinary predilection for cells lining the upper aerodigestive tract. Clinical trials based on injecting modified p53 adenoviruses (Ad-p53) have shown that it is a promising therapy and can induce apoptosis and sensitize oral cancer cells to chemo- and radiotherapy [32].

##### Targeting p53 Mutant Cells

The basis of this curative strategy is the eradication of mutant p53 protein. The effectual replication of adenovirus necessitates the deactivation of p53 function by E1B viral protein. A modified commercially available adenovirus is ONYX-015 that is unable to express E1B protein, and therefore it can encourage replication of virus and resultant cell death specifically in tumor cells harboring mutant p53 protein [33]. Clinical trials carried out in cancer patients have revealed that intravenous injection of ONYX-015 is a viable treatment option with its effectiveness increased when used in conjunction with cisplatin and 5-fluorouracil in comparison to its solitary use [34].

##### Conversion of Mutant p53 Conformation Into Wildtype

Small molecules are able to modify mutant p53 conformation into wild-type by refolding the protein, thus helping to restore its tumor suppressor function. Glycerol is a chemical chaperone reported to correct the mutant conformation of p53 into wild-type, but its usage in the clinical settings is limited due to its toxicity at higher concentrations [35, 36]. CP-31389 and APR-246 (p53 reactivation and induction of massive apoptosis, PRIMA) are small chemicals; tried on HNSCC cell lines that carry mutant p53. They are able to promote apoptosis and also inhibit proliferation by the activation of p53-dependent expression of genes, such as *BAX, CDKN1A, PMAIP1*, and *BBC3* [37]. The COTI-2 (a derivative of thiosemicarbazones supplied by Critical Outcome Technologies Inc.) can also refold the mutant p53 to restore the wild-type p53 function [38]. At present, there is no clinical data validating the efficiency of these molecules in the management of oral cancer patients and therefore further evaluation is needed.

##### Targeting p53 Inhibitors

The p53 protein function can be affected by genetic mutations or dysregulated expression of its regulators in cancer cells. The principal p53 negative regulator is MDM2 that enhances its degradation by E3 ubiquitin ligase activity. MI-219, Nutlins (*cis*-imidazoline analogs) and their modified RITA (reactivation of p53 and induction of tumor cell apoptosis) are a group of special molecules that block the binding of MDM2 to p53, thereby restoring p53 tumor suppressor function [21]. Treatments centered around these chemicals are more efficient in tumor cells harboring wild-type p53 rather than in mutant p53-harboring cells [21]. Additionally, in a subgroup of hr-HPV-associated HNSCC, the p53 function is blocked by viral E6 oncoprotein through ubiquitin-protein ligase E3A (UBE3A) also known as E6AP protein ligase and p53 is degraded through proteasome pathway and with cysteine/histidine-rich 1 (CH1) domain of p300 to prevent p53 acetylation [39]. Treatment of oral cancer cell lines with the CH1 inhibitor (CH1iB), interrupts E6 HPV 16 protein and p300 binding and consequently enhances p53 acetylation that further stimulates its transcription [40]. Additionally, Ch1iB has shown an antitumor activity in hr-HPV positive cases owing to its ability to diminish population of cancer stem cells and re-sensitizing tumor cells to cisplatin treatment [41].

##### Targeting p63 and p73

The discovery of p53 homologs, p63 and p73, has opened new areas of cancer research. Still, the role of these homologs is very intriguing and confusing in cancer progression as they encode for several proteins with similar and contrasting properties to p53. Mutant p53 with gain of function activity bind and inhibit other members of p53 family such as p63 and p73. These members can associate into homo or hetero tetramers and the mutant p53 can bind with both p63 and p73, leading to their transcriptional inhibition. This widespread functional blockage of the p53 protein family may result in higher cellular proliferation as well as cancer therapeutic resistance [42]. A proposed therapeutic approach is to disrupt the interaction between mutant p53 and p63 or p73. As a result, these homologs will become free to function. The small molecule known as reactivation of transcriptional reporter activity (RETRA) is able to induce release of p73 from mutant p53 complexes and consequently destroy the tumor. The activity of RETRA against different types of mutant p53 has been shown to be higher in cancer cells in comparison to normal cells [43].

##### Killing the Cancer Cells Containing Mutant p53

The taxane drugs such as paclitaxel are widely used to kill the cancer cells as it inhibits microtubule polymerization only in cells containing mutant p53, thereby inhibiting mitotic spindle formation and mitosis. In cells containing mutant p53, proteins associated with microtubules are heavily expressed, leading to increased microtubule polymerization [44]. Paclitaxel binding and sensitivity to microtubules is also enhanced in the presence of mutant p53 [45].

Another drug metformin, which is used to enhance the insulin sensitivity in type II diabetic patients, can selectively induce apoptosis in cells devoid of active p53. This is carried out by increased adenosine monophosphate-activated protein kinase (AMPK) activity, augmenting β-oxidation and obstructing oxidative phosphorylation. Normal cells containing wild-type p53 can modify themselves for the metformin-induced reduction in oxidative phosphorylation by accelerating glycolysis rate, on the other hand tumor cells carrying mutant p53 are incapable of adjusting their metabolism and therefore can be selectively eliminated [45].

### Retinoblastoma (*RB1*)

Retinoblastoma (*RB1*) is a tumor suppressor gene belonging to RB family and other members are *RBL1, RBL2*. Its protein product, pRb, is actively involved in the regulation of cell cycle and differentiation at the G1-S checkpoint [46]. The *RB1* gene is located on the long arm of chromosome 16, an area with high degree of loss of heterozygosity (LOH) in several tumor types including oral cancers. This LOH is reportedly present in 17% of oral cancers [47]. It has been reported that loss of *RB1* can enhance the sensitivity of tumor cells to genotoxic agents such as radiations and hormonal therapies [48]. Disruption of the *RB1* and associated signaling pathways in oral cancer can therefore be exploited to improve the efficacy of current therapies and to explore novel therapeutic strategies. Mutations in the *RB1* family members can be of germline or sporadic type however these are less frequently reported in oral cancer as compared to alterations in its signaling pathway. Frameshift mutations in 30% of HNSCCs in exons 19–22 of the RB2/p130 gene has also been reported [49] and recently a novel RB mutation 2039T>C (Ile680Thr) has been explicitly reported in oral cancer patients [50].

pRb signaling pathway is consistently abrogated in most cancers including head and neck cancers to support tumor growth and therapy failures. Members of five protein families drive this pathway forward. These are cyclin-dependent kinase inhibitor, cyclin-dependent protein kinases, D-type cyclins, pRb-family and transcription factors E2F-family (consisting of eight members and out of these, 3 members E2F1-3 preferentially bind to pRB) [51]. This pathway is crucial in the regulation of cell growth as all the members can be activated or suppressed by growth promoting or growth suppressing signals. It has been proposed that dysregulations in the RB pathway are reflected in terms of overexpression of pRb in cancers resulting in loss of tumor suppressor function. Although the exact pathogenesis for increased pRb expression and possible alterations in cancer is not clear, a number of possibilities may exist [52]. First, the overexpressed pRb may become hyperphosphorylated, which will inactivate its growth suppressing activity. pRb can exist in three forms, unphosphorylated (found in G0), hypo-phosphorylated (exists in contact-inhibited cells and in early G1) and hyperphosphorylated (inactive form in late G1, S, G2, and M phases). Therefore, in cycling cells, pRb alternates between a hypophosphorylated form, present in early G1, and hyperphosphorylated form after passage through the restriction point in late G1 and continues through S, G2 and M, phases [53]. When hypophosphorylated, pRb sequesters E2F and inhibits its activation whereas its hyperphosphorylation by cyclin-dependent kinase complexes leads to its inactivation and release of E2F. Subsequently, E2F activates its downstream target genes (*c-MYC, n-MYC, CDC-2, p21WAF-1, cyclin A, c-MYB, and EGFR*) involved in DNA synthesis, cell cycle and cell growth [54]. Second, the possibility of dysregulated pRb to support cancer progression is by dysregulated E2F1 (a member of E2Ffamily), leading to inhibition of self-mediated apoptosis, and via p53 mediated pathway [55]. Third, the binding of pRb to other proteins, such as MDM2, or certain DNA viral oncoproteins (hr-HPV E7 oncoprotein) may also override the tumor suppressor function of pRb [56].

#### Therapeutic Strategies Targeting pRb

Potential therapeutic strategies that directly target the pRb pathway comprise the revival of p16^Ink4a^ levels, blocking of Cdk4/6 kinase function, and the augmentation of E2F-mediated apoptosis [48].

### Cyclin-Dependent Kinase Inhibitor 2A (*CDKN2A*)

*CDKN2A* gene encodes two proteins p16^INK4a^ and p14^arf^, which are part of RB pathway, and they both inhibit mitosis by acting as tumor suppressors [57]. Patients harboring germline mutations in *CDKN2A* carry higher risk of oral cancer and melanoma [58]. There is also increased susceptibility to develop immunotherapy resistance in these patients [59]. There are limited number of clinical oral cancer studies that have explored the role of this particular gene in modulation of therapeutic response and this gap is yet to be filled. Hyperphosphorylated pRb release E2F which activates genes involved in progression of cell cycle such as cyclin A [60]. This mechanism is blocked by p16 which inhibits phosphorylation of pRb by coupling to the cyclin D1-CDK6/CDK4 complex. p14^arf^, which is another isoform produced by *CDKN2A* inhibits MDM2 and stabilizes p53 thereby acting as a tumor suppressor [61]. Chromosomal deletion at the 9p region that involves the *CDKN2A* gene locus has been recognized as the most common chromosomal aberration. In addition, there are reports of somatic mutations in p16 in 21% of oral cancer samples by analyzing the TCGA database [62]. It has also been proposed that *CDKN2A* chromosomal abnormalities are frequently linked with cyclin D1 gene overexpression in oral cancer [63]. This supports the fact that high p16 expression has been linked to favorable prognosis in OSCC while overexpression of cyclin D1 is linked to poor prognosis. It is uncertain whether genetic modifications at the 9p21 locus (*CDKN2A*) by themselves are enough to initiate carcinogenesis, as these have also been identified in benign cases [62]. Moreover, genetic modifications and p16 inactivity commonly occurs through epigenetic silencing such as promoter hypermethylation reported in 80% of oral cancer [64].

#### Therapeutic Strategies Targeting p16

*In vitro* studies with the demethylating agent, 5'-azacitidine, have revealed an increase in p16 levels. The adenovirus-mediated gene therapy for p16 has also been tested in oral cancer. Furthermore, CDK4 blockers are presently in phase I clinical trials for solid tumors and hematologic malignancies but no studies have been conducted on oral cancer [65].

### Epidermal Growth Factor Receptor (EGFR)

Epidermal growth factor receptor (EGFR/ErbB1/HER1) is proposed as a proto-oncogene that belongs to tyrosine kinase receptor family; other members include ErbB2/HER2/Neu, ErbB3/HER3 and ErbB4/HER4. The EGFR activation requires binding of the ligand to its extracellular domain (ECD), whereas its cellular effects depend on the activation of its cytoplasmic tyrosine kinase domain [66]. Similar ligands are also released by tumor cells during cancer progression and contribute in autocrine and paracrine effects. Stimulation of EGFR may further activates PI3K/Akt, Ras/Raf/MAPK, PLC/PKC or JAK/STAT pathways involved in diverse cellular processes such as metabolism, growth, survival, apoptosis, and differentiation [67].

*EGFR* is an extremely polymorphic and mutation-prone gene, with more than 1,200 single nucleotide polymorphisms (SNPs) reported in the literature [68]. Somatic mutations in the EGFR are reported in over 90% of HNSCC causing persistently raised or continued EGFR signaling. Such irregular signaling is linked not only to increased growth and reduced apoptosis in tumor cells, but also induces angiogenesis and metastasis with poor prognosis [69]. A recent analysis revealed that about 2.8% of oral cancers carry mutations in the tyrosine kinase domain of EGFR [70]. Mutations in EGFR in HNSCC are scattered from exons 18 to 21. Of all the EGFR mutations, in-frame deletions in exon 19 were the most common followed by missense mutation L858R and T790M in exon 20. The EGFR mutational landscape in HNSCC has not been sufficiently evaluated globally as is reflected from very few studies on the subject [70]. A subcategory of HNSCC shows a shortened EGFR splice variant, known as EGFRvIII, in which the ligand-binding domain is modified as a result of deletion of 6–273 residues. This modification continuously stimulates the receptor even in the absence of EGF and TGF which leads to increased cell proliferation, survival, motility and invasion [71].

In addition to somatic changes, patients can show overexpression of EGFR which is associated with higher locoregional failure, suggesting that abnormal EGFR signaling can contribute to chemotherapy, radiotherapy and immunotherapy resistance [72]. There are several possible mechanisms for such resistance. First, radiations given during radiotherapy imitate ligand–receptor interaction by inducing EGFR autophosphorylation, which stimulates PI3 kinase and Ras pathways thereby supporting growth and survival of tumor cells, and eventually leading to therapy failure. Second, after Ras stimulation, the downstream MAP kinase mediates the sustained production of amphiregulin, EGF and TGF monomers that make an autocrine circuit, thus facilitating hyperproliferation [72]. Third, the overexpressed EGFR may also activate radiation-induced DNA double strand breaks via ataxia telangiectasia mutated (ATM) gene regulation that plays a crucial role in phosphorylation of the EGFR–DNA-PK-Ku complex which intermediates DSB DNA repair. These EGFR mediated signaling cascades together with the hyperactivation of the DNA repair system may lead to the therapy failure observed in cancer patients [73].

#### Therapeutic Strategies Targeting EGFR

##### Antibodies Targeting EGFR

**Figure 3:**
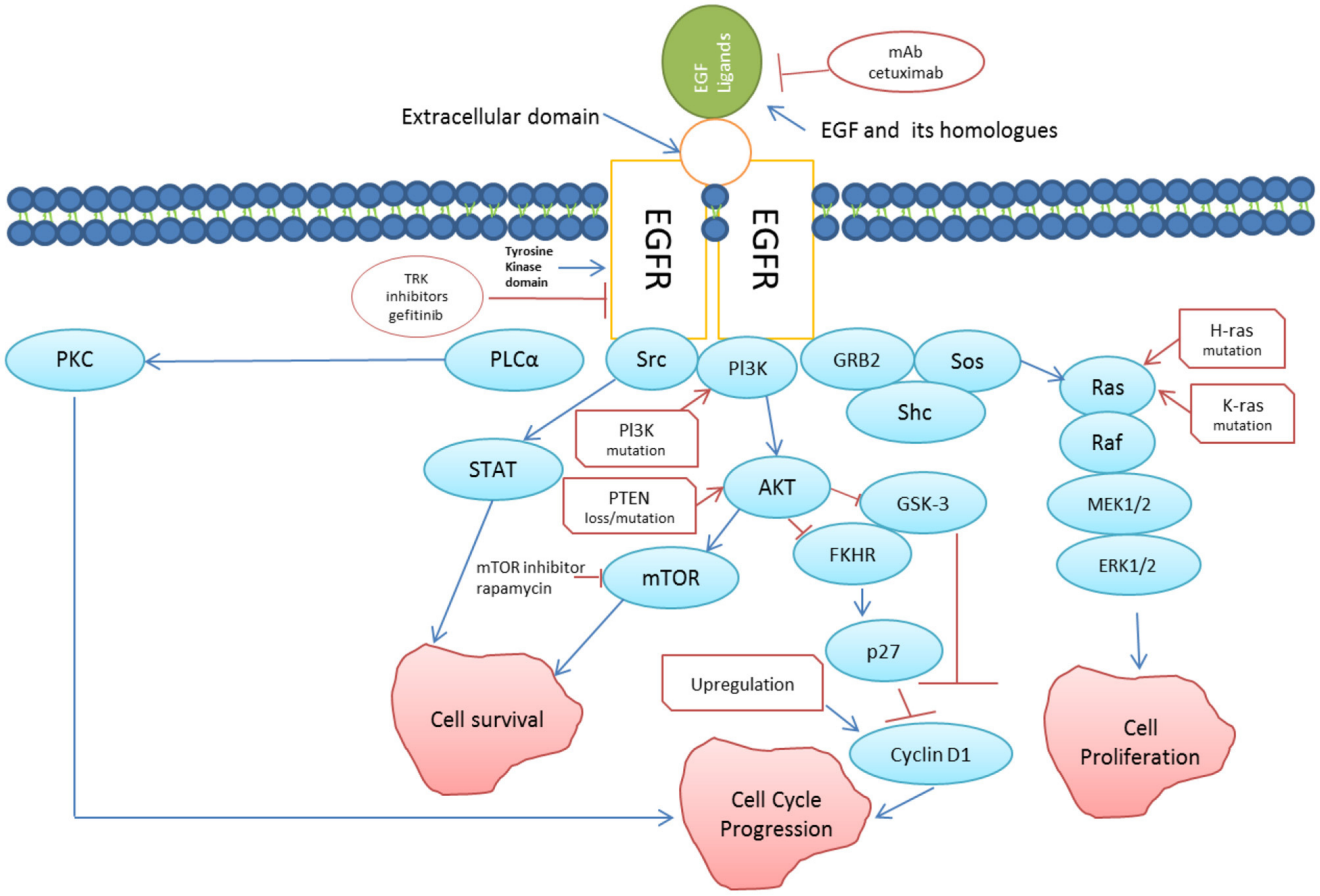
EGFR signaling pathway (redrawn from [75]). Binding of EGF (and its homologs) to EFGR triggers directly or indirectly cascades of reactions leading to cell survival and cell proliferation. Somatic mutations in EGFR leading to persistent activation is linked to increased growth, reduced apoptosis, increased angiogenesis and metastasis, which are the key features of cancer progression. Two approaches using mAb against EGFR such as cetuximab, and tyrosine kinase inhibitors to suppress anomalous EGFR activation in cancer cells has been shown in the figure.

One of the main monoclonal antibodies targeting EGFR is known as Cetuximab [74] (Figure 3). There are other anti-EGFR antibodies under investigation for use in combination with chemo- and radio-therapy for oral cancer treatment such as zalutumumab, panitumumab and nimotuzumab [76]. Cetuximab, a chimeric IgG1 mAb, binds to the ECD of EGFR, inhibits normal receptor interaction, thereby preventing the activation of downstream signaling pathways [74]. Panitumumab enhances radio-sensitivity via radiation-mediated DNA damage and inhibiting the translocation of EGFR to the nucleus. Presently, radiotherapy in combination with panitumumab is undergoing phase III clinical trial [77]. Of all therapeutic antibodies available, cetuximab is the most effective in enhancing radio-sensitivity in high-EGFR expressing cells [78]. Some cancer patients may develop therapeutic resistance to anti-EGFR therapy in the presence of EGFRvIII variant due to reduced affinity of the mAbs raised against the wild-type EGFR [79].

##### EGFR Targeting Through TRAIL and Smac Mimetics Molecules

Two classes of new therapeutic agents directed against molecules involved in apoptosis pathway have been developed that can be used in combination with anti-EGFR therapy in oral cancers to overcome EGFR polymorphism based therapeutic resistance. The first is the tumor necrosis factor-related apoptosis-inducing ligand (TRAIL), which can induce cell death by binding to TRAIL-R1/R2 receptor [80]. The second class of anticancer agents are composed of Smac mimetics (SM), which simulate the function of pro-apoptotic mitochondrial protein Smac/Diablo. In response to a stress signal, Smac/Diablo comes out in the cytoplasm and blocks the anti-apoptotic function of inhibitor of apoptosis proteins [81]. A study analyzing the sensitivity to TRAIL and SM management in oral cancer cell lines showed that the two molecules are extremely active in promoting tumor cell apoptosis. Furthermore, expression of caspase 8 and tumor necrosis factor alpha (TNF-α) were recognized as biomarkers for the respective evaluation of TRAIL and SM sensitivity [82].

##### EGFR Tyrosine Kinase Inhibitors

Tyrosine kinase inhibitors (TKIs) are small molecules that also target the kinase domain of EGFR, preventing its autophosphorylation and subsequent activation. TKIs include lapatinib (Tyverb), afatinib (Giotrif), brigatinib (Alunbrig), erlotinib (Tarceva) and gefitinib (Iressa) with only the last two being well studied [83, 84]. Laboratory studies have shown that gefitinib inhibits cell proliferation, decreases cell survival and enhances tumor cell therapeutic sensitivity [85]. Additionally, encouraging results were obtained in clinical studies by using gefitinib in combination with VEGFR blockers signifying its possible use as a new therapeutic agent [68]. It is noteworthy that the clinical trials combining gefitinib with chemo- and/or radiotherapy have not established significant improvement compared with conventional therapy. Combination of erlotinib targeting EGFR along with VEGF inhibitors has also shown desirable results in clinical phase I and II trials in metastatic and recurrent oral cancer [86]. Afatinib and Lapatinib are orally administered well tolerated EGFR and HER2 inhibitors in cancer patients [87]. Many patients may develop resistance to TKIs, which can be attributed to T790M mutation in EGFR or co-mutations in cMET, a proto-oncogene which has predilection for hepatocyte growth factor (HGF). Results of phase II clinical trials are encouraging for brigatinib which can target the T790M mutation, and it is acknowledged as a “Breakthrough Therapy” by the FDA [84].

##### Vaccine Targeting EGF

The CIMAvax-EGF vaccine comprises of a chemical conjugate of EGF with the p64 protein of meningitis B bacteria. The vaccine induces antibodies against EGF, which blocks EGF-EGFR interaction and inhibits EGFR autophosphorylation. This strategy as a cancer therapy against NSCLC is in use in Cuba, however, there are no ongoing clinical trials for its usage on oral cancer patients [88].

### RAS

Rat sarcoma virus (*RAS*) is a protooncogene and its three family members are Harvey-Ras (H-Ras), Neuroblastoma Ras (N-Ras) and Kristen Ras (K-Ras having isoforms A and B) [89]. This family encodes Ras proteins having inherent guanosine triphosphatase (GTPase) activity and stimulates downstream signaling cascade via Raf-MEK-ERK, PI3K/AKT or c-Jun N-terminal kinase (JNK) pathways involved in cellular proliferation, migration, adhesion and differentiation after growth factor stimulation such as EGFR [90]. This gene and its signaling pathway is frequently mutated in oral cancer and mostly the mutations (T81C, Q61R, G12V and G13R) are reported in H-Ras [91]. These mutations are mostly reported in smokers, betel quid chewers and also show ethnic variations [90]. The studies have highlighted the role of H-Ras mutations in treatment failure or development of resistance to EGFR tyrosine kinase inhibitors such as cetuximab and erlotinib in oral cancer patients [92]. The proposed mechanism of therapeutic resistance to EGFR TKIs include constant stimulation of downstream signaling pathways by mutated RAS gene in oral cancer via special group of genes such as *CCND1, c-MYC, BCL-XL* and *BCL-2* [93].

#### Therapeutic Strategies Targeting Ras

To overcome the therapeutic resistance conferred by RAS mutations to the EGFR inhibitors in oral cancer patients, the different approaches reported so far are; First, farnesyltransferase inhibitors (FTIs) such as Tipifarnib that compete with Ras and suppresses its activity. But it also leads to the inhibition of other non-targeted proteins such as centromeric proteins, CENP [94]; Second, blocking both PI3K/Akt and MAPK pathways via MEK and Akt inhibitors. Third, blocking post-translational modification of Ras by ICMT1 (isoprenylcysteine carboxyl methyltransferase-1) and RCE1 (ras-converting enzyme 1) but this approach was not affective [89].

### AurkA/B

Aurora kinases A and B (AurkA and AurkB) are extremely preserved serine/threonine kinases that perform an indispensable and discrete function in mitosis. AurkA is essential for mitotic spindle assembly and is located to centrosomes at spindle ends throughout the prophase up to metaphase [95]. Additionally, higher expression of AurkA leads to atypical centrosome numbers and the generation of aneuploidy which can lead to cell proliferation, tumor progression, and metastasis. It has been reported that overexpression of EGFR and AurkA in tumor tissues is a risk factor associated with poor disease-free survival and therapy resistance [96]. Additionally, AurkA/AurkB and EGFR have the same downstream signaling pathways, rendering them both as a potential therapeutic target in oral cancer. AurkA is physically or functionally related to many other key targets involved in tumourigenesis, with over 60 interacting partners including NMyc, IkBa, AKT, RalA, p53, TPX2, NEDD9, survivin etc [97].

#### Therapeutic Strategies Targeting Aurora Kinases

The combination of cetuximab and AurkA/B inhibitors can improve treatment efficiency in any EGFR polymorphism-based therapeutic resistance in cancer cells [96]. Aurora-A and -B targeting agents (ZM447439, AZD1152 and ENMD2076), pan-Aurora-inhibitors (AT9283 and AMG900), the Aurora-B/C inhibitor (GSK1070916A) and Aurora-A-specific agent (MLN8237/alisertib), are under clinical trials for solid tumor (ovarian, breast and colon) treatment [98].

### Notch

Notch family consists of four members, Notch 1 - 4. These are small proteins involved in proliferation and differentiation and self-renewal [99]. The function of Notch in tumourigenesis is intriguing and understudied, it is reported to be either oncogenic or anti-proliferative, but most of the studies have reported it to be oncogenic. Although Notch is upregulated in oral cancer [100], some studies have suggested that Notch stimulation limits proliferation and supports differentiation [99]. It is also an important signaling pathway involved in developing chemo-resistance in tumor cells through maintaining cancer stem cell population, induction of epithelial mesenchymal transition (EMT), DDR hyperactivation and angiogenesis. Down-regulation of the Notch can induce drug sensitivity and overcome therapy resistance [99, 100]. It has been described that the average mutational rate for Notch1 in oral cancer was 12.67% compared to 4% for Notch2 and Notch3. The common Notch1 mutations reported in oral cancer are missense that occur on or near the ligand binding domain (EGF repeats) or the ankyrin domains [62].

#### Therapeutic Strategies Targeting Notch Signaling Pathway

To target Notch signaling cascade, drugs under investigation are, a: γ-secretase inhibitors (GSI) that can inhibit the ligand-induced processing of Notch receptors; b: mAb against Notch that binds the extracellular domain of Notch receptor; c: A Dis-integrin And Metalloproteinase, ADAM17, inhibitor that stops the initial step of ligand-stimulated processing of Notch receptors; and d: Notch Intracellular Domain (NICD) protein-protein-interaction blockers that inhibit the NICD-mediated activation of Notch effector genes [101, 102]. Currently few studies on xenograft oral cancer models are employing the above mentioned strategies but no clinical outcome has been reported [101, 103].

### PTEN/mTOR/AKT1/PIK3CA

Current molecular depiction has shown that in oral cancer, PTEN/mTOR/AKT/PI3K appears to be the repeatedly dysregulated pathway and is related to chemo- and radiotherapy resistance via autophagy stimulation, angiogenesis and co-activation of linked signaling pathways [104]. Normally this pathway is vital for regulating the cell cycle and hence affects cellular quiescence, proliferation and cancer progression [105]. Autophagy is a complex protective catabolic process in a cell to induce self -digestion of organelles to maintain homeostasis and cell population [106]. Persistent autophagic signals usually lead to apoptosis and protect from carcinogenesis under normal circumstances. However, in well-established growing tumors, autophagy helps to compensate for metabolic stresses such as ischemia and nutrient deprivation by providing energy to the cancer cells through degradation and utilization of own proteins [107]. This stress tolerance mechanism in cancer cells brought through autophagy helps them to resist different anticancer agents. Inhibitors of mTOR, such as PTEN, induce autophagy whereas oncogenes that stimulate mTOR such as Ras, PI3K and AKT suppress autophagy [108]. Interestingly, genetic and microenvironmental factors influence the way cancer cells exploit autophagy for their own survival [106, 109].

Phosphoinositide 3-kinases (PI3K) are a class of enzymes playing a key role in cellular survival, growth and differentiation. It is triggered by RTK, such as EGFR. PI3K activation phosphorylates and activates protein kinase B (PKB or AKT), which is then transported to the plasma membrane [110]. AKT can have different downstream effects such as triggering the mammalian target of rapamycin (mTOR) complexes (mTORC1 and mTORC2), which can activate transcription of p70 and other signaling molecules of the PI3K pathway, including serine/threonine protein kinase SGK146. This activation further leads to a surge in protein synthesis and cell proliferation [111]. There are numerous stimuli that augment the PI3K/AKT pathway including IGF-1, EGF, insulin and sonic hedgehog (SHH) [112]. This pathway is antagonized by various factors including phosphatase and tensin homolog (PTEN). The PTEN protein behaves as a tumor suppressor through the action of its phosphatase protein product to dephosphorylate phosphatidylinositol-trisphosphate (PIP3) to PIP2 [113]. This dephosphorylation is significant because it results in inhibition of the PKB/AKT signaling pathway, which is crucial in controlling the cellular functions such as cell growth, survival, and migration. During tumor development, mutations and alterations in PTEN/PI3K/AKT/mTOR pathway lead to increased cell proliferation and reduced cell death [114]. These mutations are reported to confer drug resistance in oral cancer treatment. The reported mechanism behind drug resistance is attributed to altered *MDR-1* gene activation and prolonged cell survival. Most common mutational sites are in exon 9 and exon 20 of PIK3CA in oral cancer. Frequent mutations in this gene include E542K, E545K, H1047R, H1047Y, and H1048Q [115].

#### Therapeutic Strategies Targeting PTEN/PI3K/AKT/mTOR

Clinical trials have evaluated the value of targeting PTEN/PI3K/AKT/mTOR pathway with various drugs (Figure 3), including everolimus, idelalisib, rapamycin, temsirolimus, wortmannin and bortezomib with positive results [116]. Resistance to mTOR blockers has also been reported in oral cancer management, but the mechanisms responsible for this therapeutic resistance are still being explored. A likely feedback link between ERK/MAPK and AKT signaling by mTOR blockage may be responsible for cancer cells survival [117]. Targeting mTOR along with EGFR, therefore may block the upstream stimulation of ERK and AKT signaling, consequently helping to overcome this type of resistance [118].

### c-MET

During epithelial cancer progression, tumor cells lose their specific markers such as E-cadherin, cytokeratins and express mesenchymal proteins such as vimentin, fibronectin and N-cadherins to migrate to distant areas of the body. This process is called epithelial mesenchymal transition EMT [119]. After migrating to distant areas of the body, these mesenchymal cancer cells revert back to express epithelial markers through a process called Mesenchymal epithelial transition (MET) [120]. The *c-MET* gene encodes the “c-MET tyrosine kinase” which induces metastasis and tumor invasion [121], and may play an important role in therapeutic resistance [122]. Mutations in c-MET are not common in oral cancers (2–13%), but higher MET copy number and increased expression of its ligand, HGF, are frequent [123]. In oral cancer patients, c-Met stimulation is associated with poor outcome and reduced survival. As c-Met and EGFR have the same downstream pathways, the activation of MET-HGFR cascade may identify a therapeutic target in oral cancer especially in patients with resistance to EGFR-targeted therapies [124]. It is interesting to note that studies on the inhibition of EGFR along with c-Met have shown improved anti-tumor activity and re-sensitization of cells to EGFR targeted therapies [125].

#### Therapeutic Strategies Targeting c-MET

Capmatinib is a c-Met inhibitor that has shown anti-cancer activity in murine models. A phase I trial evaluating capmatinib efficacy in advance solid tumors has been conducted and the results are awaited [126]. Ficlatuzumab, an antibody that targets the HGFR/c-Met axis, is also being tried in clinical settings along with cetuximab for oral cancer management [127]. An anti-CD44 antibody, RG7356, targeting c-Met (a co-receptor of CD44) by modifying MAPK pathway is being currently investigated [128].

### Jenus-Activated Kinases (JAK)/Signal Transducer and Activator of Transcription (STAT)

Dysregulation of the signal transducer and activator of transcription (STAT) family has been described in both HPV positive and negative oral cancer. Higher levels of STAT3 and its effectors are proposed to intensify the metastatic potential of oral cancer, and increase its resistance to chemo-, radio- and EGFR-directed therapies [129]. STAT3 pathway is reported to be immunosuppressive and may shield tumor cells from identification and destruction by cytotoxic T lymphocytes. This is obtained by eliciting the production of cytokines, including IL-10, IL-6, TGF-β and VEGF [130]. STAT3 pathway is triggered by the upstream effects of the IL-6 cytokine receptor family, RTK such as VEGFR, EGFR, Src family kinases (SFK) and Jenus-activated kinases (JAK). Upon stimulation, nuclear phosphorylated-STAT3 activates target genes with pro-survival factors, such as survivin, cyclin D1 and BCL-XL [131, 132].

#### Therapeutic Strategies Targeting JAK/STAT

For STAT3 targeting, ruxolitinib is a FDA approved JAK inhibitor for myelofibrosis [133]. Presently, a clinical study is underway with the aim of testing the efficiency of ruxolitinib in oral cancer. AZD9150, a fabricated anti-sense oligonucleotide affecting STAT3 translation, has validated anti-cancer function in xenograft models [134]. Currently, it is being tested for metastatic oral cancers both alone or in combination with MED14736, a drug which blocks binding of programmed cell death protein 1 (PD1) to its ligand [132].

### MAPK Pathway

The mitogen-activated protein kinase (MAPK) pathway affects and controls the levels of different proteins intricately involved in cell growth, maturation, programmed cell death, angiogenesis and metastasis [135]. It includes four sub-mechanisms, of which the Erk1/2 pathway is considered of utmost importance. After binding of ligands (such as EGF) to their receptors, a signaling complex stimulates mitogen-activated protein kinase 3 (MAPK3/p44MAPK/Erk1) and Erk2 (MAPK1) that dissociates from the Ras-Raf-MEK-Erk1/2 complex and consequently different structural proteins, transcription factors including AP-1, NF-κB, c Myc and ETS-1 and kinases (RSK1-4, MNKs, MSKs, MK2/3/5) are phosphorylated [136]. Mutations in the members of MAPK signaling pathway have been reported to modulate the response to chemotherapies and targeted therapies [137].

#### Therapeutic Strategies Targeting MAPK

Inhibitors of MEK, an upstream kinase to MAPK, such as trametinib, are presently being studied in clinical settings for oral cancer treatment [138].

### Fas/Fas Ligand (FasL)/MMP

FasL and its receptors (Fas, CD95) are important members of tumor necrosis factor (TNF) family, contributing to the immune regulations. The collaboration between FasL and Fas initiates the process of apoptosis [139]. FasL is overexpressed in numerous tumors, including oral cancer, and is associated with providing anti-apoptotic potential [140, 141]. Cancer cells enhance their survival chances during tumor development by diminishing their response to Fas-mediated apoptosis. Possible modes for Fas desensitization include reduced Fas protein and inhibition of binding of the stimulated receptor to the soluble Fas ligand (sFasL), or via both mechanisms. Chemotherapeutic agents leading to upregulation of matrix metalloproteinase 7 (MMP7), that causes the production of sFasL and therefore FAS/FASL mediated apoptosis is downregulated leading to therapy failure [142]. MMPs have the basic function to break down extracellular matrix (ECM) by degrading macromolecules including types I, II, IV, and V collagens, fibronectin and proteoglycan. High level of MMP7 expression accelerates cancer invasion and angiogenesis by cleavage of ECM and connective tissues. During cancer progression, MMP7 degrades cell surface proteins, promotes adhesion of cancer cells, and consequently promotes tumor metastasis [140]. A number of reports have suggested that polymorphisms in MMP3 and MMP7 are important risk factor for therapeutic resistance in cancer. For MMP7, two polymorphisms have been reported, 181A → G and −153C → T while in MMP3, the insertion or deletion of an adenine at position −1612 (−1612insA) demarcates two alleles 5A or 6A. The 6A allele is associated with reduced transcription compared with 5A. An important positive association between MMP3 6A isoform and chemotherapy sensitivity in French oral cancer patients has been reported with subjects having 6A allele responding better to 5-FU-cisplatin combination treatment [143].

#### Therapeutic Strategies Targeting Fas/Fas Ligand (FasL)/MMP

MMPs are favorable targets for cancer treatment due to their higher expression in cancer tissues and their capability to destroy all constituents of the ECM. Synthetic metalloproteinase inhibitors (MPI) have been developed and undergoing human clinical trials for pancreatic and lung cancer but the outcomes of these studies so far are not promising [144].

### FAT1

In humans, there are four members of FAT family, these are FAT1 - 4 that encodes proteins Fat1−4, respectively. These proteins are involved in intercellular adhesion, morphogenesis, migration and interaction with ECM and different signaling cascades [145]. FAT1 is reported to be active in conferring cisplatin based chemotherapy resistance in oral cancer [146]. Limited studies are available on the role of this molecule in oral cancer and have shown conflicting results. These proteins are reported to be tumor suppressors via inhibition of Yes-associated protein (YAP) 1 and suppress cell growth and metastasis in oral cancer [147, 148], conversely these are also shown to be proto-oncogenic and play a role in metastasis and apoptotic suppression in different carcinomas [149]. Oncogenic potential of FAT1 may be attributed to mutational changes (nonsense, missense, and frameshift) in the gene that render it oncogenic. Studies have shown that FAT1 can be targeted to re-sensitize cells to cisplatin chemotherapy via downregulating LRP5/WNT2/GSS signaling and increased oxidative stress [146].

#### Therapeutic Strategies Targeting FAT1

Treatment options to target FAT1 includes FAT1 mAb 198.3. It is being investigated for colon cancer treatment [150]. No study has so far been reported on its use in oral cancer [103].

### CASP-8

*CASP-8* gene is primarily a tumor suppressor gene encoding procaspase-8 protein found on chromosome 2q33-34 [151]. Gene product is a cysteine endoprotease that is a key factor for functionality of apoptotic pathway when triggered by death signals [152]. Therefore, it is crucially important in tumor progression and resistance in chemo- and radiotherapy induced cancer cell death via apoptotic pathways [153]. Resistance to cytotoxic drugs due to apoptotic suppression is attributed to different mechanisms such as somatic changes (gene deletions, promoter methylation) in caspase-8, its sequestration by Bcl-2 or overexpression of its inhibitors such as FLIP (FLICE-like Inhibitory Protein). Therefore, this molecule can be targeted to restore treatment sensitivity [153]. Recently, it has been proposed that a specific mutation Gly325Ala in caspase8 gene enables it to induce NF-κB-mediated expression of different cytokines and angiogenesis. Therefore cancer cells harboring such mutation has the higher potential for growth and progression [154]. Studies have also shown that mutations in *CASP-8* namely R417X (C>T), R218Q (G>A), G310D (G>A), G310D (G>A), D200fs (del TATT frameshift deletion at exon 4), E204X (G>T), Q225X (C>T), T258fs (C>T) and L428Q (T>A) affect functions of *CASP-8* and these are positively correlated with cancer progression [155]. However, one single nucleotide polymorphism SNP on the *CASP-8* promoter (−652 6 bp ins/del) is reported to negatively co-relate with cancer progression [156]. *CASP-8* is also active in other cellular mechanisms such as autophagy, cell adhesion, migration and endosomal trafficking. These functions are reported to be hijacked by cancer cells to progress and invade multiple sites. Based on these assumptions, it is now believed that caspase-8 can behave as tumor suppressor or proto-oncogene. Therefore, new synchronized treatment strategies that raise caspase-8 level while inducing its apoptotic function should be explored [152].

#### Therapeutic Strategies Targeting CASP-8

Expression of casp-8 can be enhanced via chemical compounds such as indolones, MX-2060 (by Maxim pharmaceutical company, Kenya) and dichlorobenzyl carbamates to induce apoptosis in tumor cell. Currently no clinical trials have been reported for these chemicals in oral cancer [157].

## Epigenetic Changes

Epigenetic changes involve modifications in chromosomal structure such as hypermethylation of CpG island and alterations in histone [158]. These changes are reported in nearly all tumors and induce tumor growth and confer therapeutic resistance. Many studies on oral cancer have analyzed effect of promoter methylation on tumor suppressor genes. Abnormal methylation of *CYGB, CCNA1*, and *CDKN2A* and *CDKN2B* has been linked to different precancerous, cancerous and salivary gland carcinomas [159]. Whole-exome sequencing has identified histone methyl-transferases MLL2 and EZH2 (active in preserving chromosomal structure and transcriptional stimulation) as the most commonly mutated genes in cancer [40]. In another TCGA data analysis, the histone alteration genes MLL2, MLL3, and NSD1 were highlighted as commonly mutated in HNSCC with a rate of 17.9, 7.3, and 10.6%, respectively [160]. Epigenetic changes can potently modify many transcriptional outputs and variations in the levels of a variety of genes [160].

### Therapeutic Strategies Targeting Epigenetic Changes

Exploring the epigenetic mechanisms in cells has opened novel prospects for cancer management. The main goal is to disrupt transcriptional mechanism mediating the carcinogenesis by targeting epigenetic enzymes and thus therapeutic benefits can be derived [40]. Inhibitors of DNA methyl-transferases (DNMT) such as decitabine and 5'-azacitidine or histone deacetylases (HDAC), including romidepsin or vorinostat are FDA approved epigenetic cancer drugs [161]. Recently, the identification of loss of function alterations in epigenetic proteins has added huge difficulty in recognizing effective drug targets. The fact that epigenetic mechanisms are frequently regulated by opposite groups of enzymes or pathways may be a likely answer to this problem [62].

## DNA Damage Repair System

The DNA damage repair (DDR) system protects the genetic material from any aberrations to maintain total integrity. DNA can be impaired by exogenous factors such as chemicals and radiations as well as endogenous factors such as reactive oxygen species (ROS) [162]. A variety of proteins intricately involved in DDR are related with chemo- and radio-resistance in oral cancer. If only a single strand of a double helical DNA is damaged, it is called single-strand DNA damage (SSD), and in such cases the second strand can be utilized as a prototype to direct the rectification of the impaired strand. Base excision repair (BER), nucleotide excision repair (NER) and mismatch repair (MMR) are different repair mechanisms that are used in repairing SSDs [163]. Double-strand breaks [75], comprising of damage in both DNA strands are dangerous to the cell as they can lead to genetic relocations. Three possible pathways repair DSBs; 1, non-homologous end joining (NHEJ), 2, microhomology-mediated end joining (MMEJ); and 3, homologous recombination (HR) [164]. The major DSB repair mechanism is NHEJ, in which a platform is provided by the Ku 70/80 protein that interacts with DSBs and associated proteins such as DNA-PKcs, BRCA1, APLF, and PAXX involved in the repair. In the next phase, DNA-PKcs associates the kinase with Ku, DNA, BRCA1, PARP1, and Artemis [165]. This transitory framework assists restoration and is based on XRCC4/XLF filaments that link Ku bound to DSB terminals. DNA ligase IV bound to XRCC4 then dissociates the framework, bringing Ligase IV close to the DNA ends and joining them, thereby completing the repair [166]. Multiple proteins interacting in DDR are linked to chemo- and radio-resistance in oral cancer. Since cancer cells predominantly use NHEJ repair mechanisms to repair their damaged DNA [167], exploring new targets in this repair pathway may help to increase disease control and overall patient survival [72].

Another mechanism by which DNA recognizes and repairs invalid insertion, deletion, and mis-incorporation of bases arising during replication is the DNA mismatch repair (MMR) system [168]. MMR failures due to mutations in associated proteins (such as MSH2/6, MLH1/2 and PMS2) affect the genomic stability and leads to the formation of small unstable repeated sequences of DNA called microsatellites, which supports oncogenesis. The involvement of MSI in oral cancer has been less clearly revealed. However, few studies have shown that direct mutation or deletion of MLH1/MSH2 are not frequent in oral cancer. The principal mechanism of MMR dysfunction is via epigenetic changes such as promoter hypermethylation rather than direct mutation. MLH1 promoter hypermethylation has been described in 8–69% of oral cancer samples [169].

### Therapeutic Strategies Targeting DNA Damage Repair System

**Figure 4:**
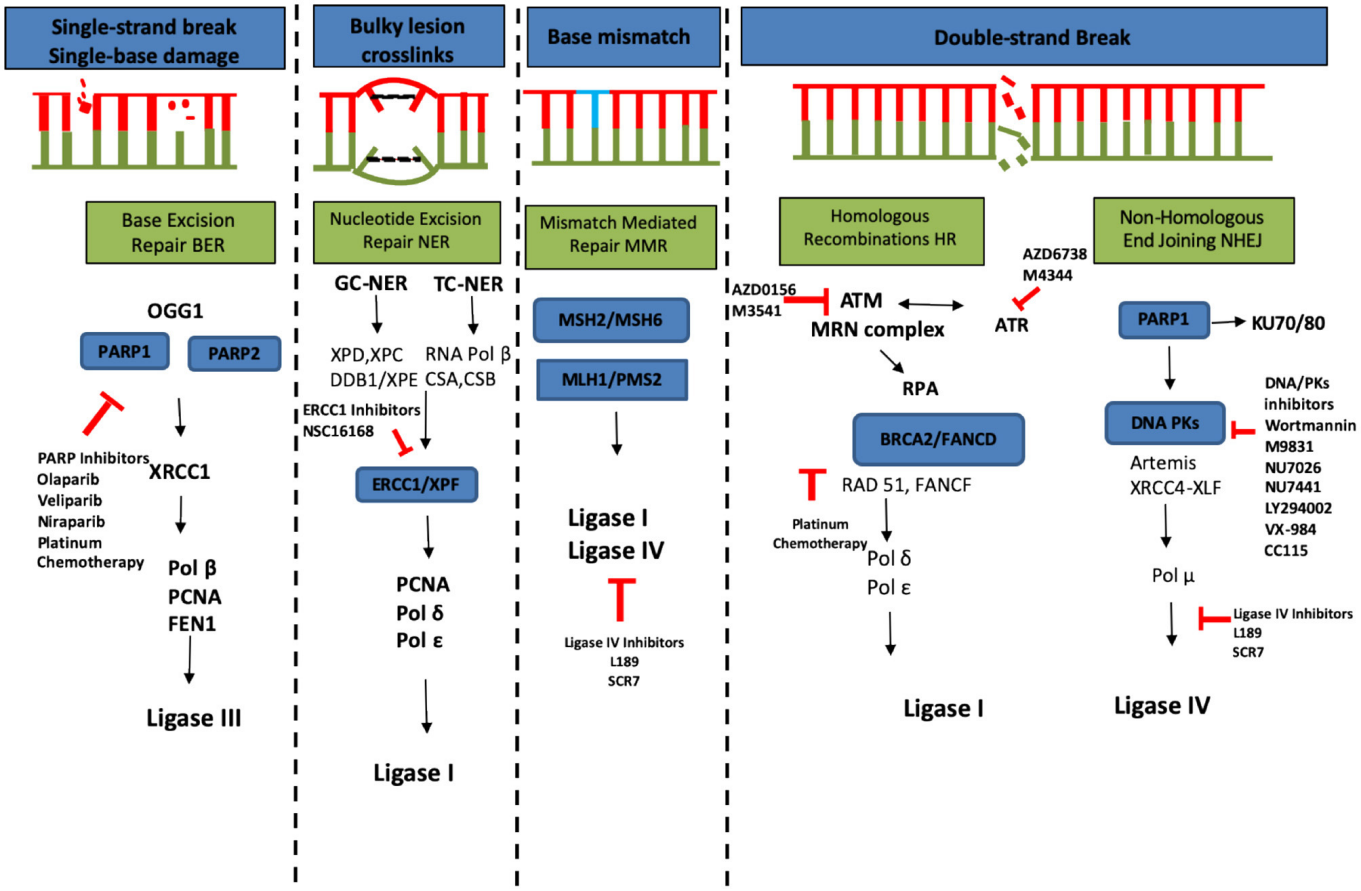
DNA damage responses and repair pathways (redrawn from [171]). Chemo- and radiotherapy used to treat cancer can cause a variety of damage to the cellular DNA including single-strand break caused by single base damage, crosslinking of bases, mismatch of bases and double strand breaks. This causes hyperactivation of DNA repair machinery which is an important tool utilized by cancer cells to compensate for the damage. Multiple ongoing strategies to block the DNA repair and thus re-sensitize cancer cells to different therapeutic agents are summarized in this figure (red color).

The repair of DSBs via NHEJ has the potential to sensitize tumor cells to chemo- and radiotherapy [170]. Different molecules targeting DDR pathway proteins are summarized in Figure 4. Oncogenesis due to MMR failure and hyperactive DNA repair pathways carry high genomic instability and generate neoantigens, therefore in such cases immune checkpoint inhibitors, such as those directing against PD-1 (pembrolizumab), or the ligand PD-L1 (MPDL3280A) and cytotoxic T-lymphocyte-associated protein 4 (CTLA-4) (ipilimumab), are more beneficial [172].

## Micro RNAs

A family of small non-coding RNAs termed microRNAs are endogenous 17–25 nucleotides in length, proposed to post-transcriptionally control about 30% of human genome mainly via partial complimentary binding to mRNA of targeted genes leading to its degradation, destabilization or translational repression [173]. Hence by modifying the mRNA they can regulate expression of multiple genes [174]. The role of miRNA in different cancers is comprehensively investigated in recent years. The significance of miRNA in tumor development and progression has been strongly advocated by their associated altered expressions, repeated amplifications and genomic deletions during cancer initiation and progression. Two types of cancer-related miRNA are found, oncogenic or tumor suppressor miRNA [175]. Extensive studies on miRNA have revealed that they can be used as therapeutic agents and their role as a marker of disease progression and predictor of therapy response is also now well-established [87]. Some of the miRNAs that are implicated in oral cancer development/progression include miR-31, miR-34, miR-375, miR138, miR-203, miR-200c, miR-222, miR-377, miR-30a-5p, miR-155, miR373-3P, miR-218, and miR455-5p [173].

### Therapeutic Strategies Targeting Micro RNAs

Evidence has suggested the possibility of observing variations in miRNA expression earlier or during therapeutic period can estimate the response to specific treatments [176]. Modulation of dysregulated miRNA by molecules that substitute downregulated miRNA or use of blockers which bind upregulated miRNA may be of future applications [173]. Currently, only one clinical study is available in liver cancer patients with MRX34, a molecule imitating miR-34. No clinical trials have been reported for oral cancer [177].

## Cancer Stem Cells

**Figure 5:**
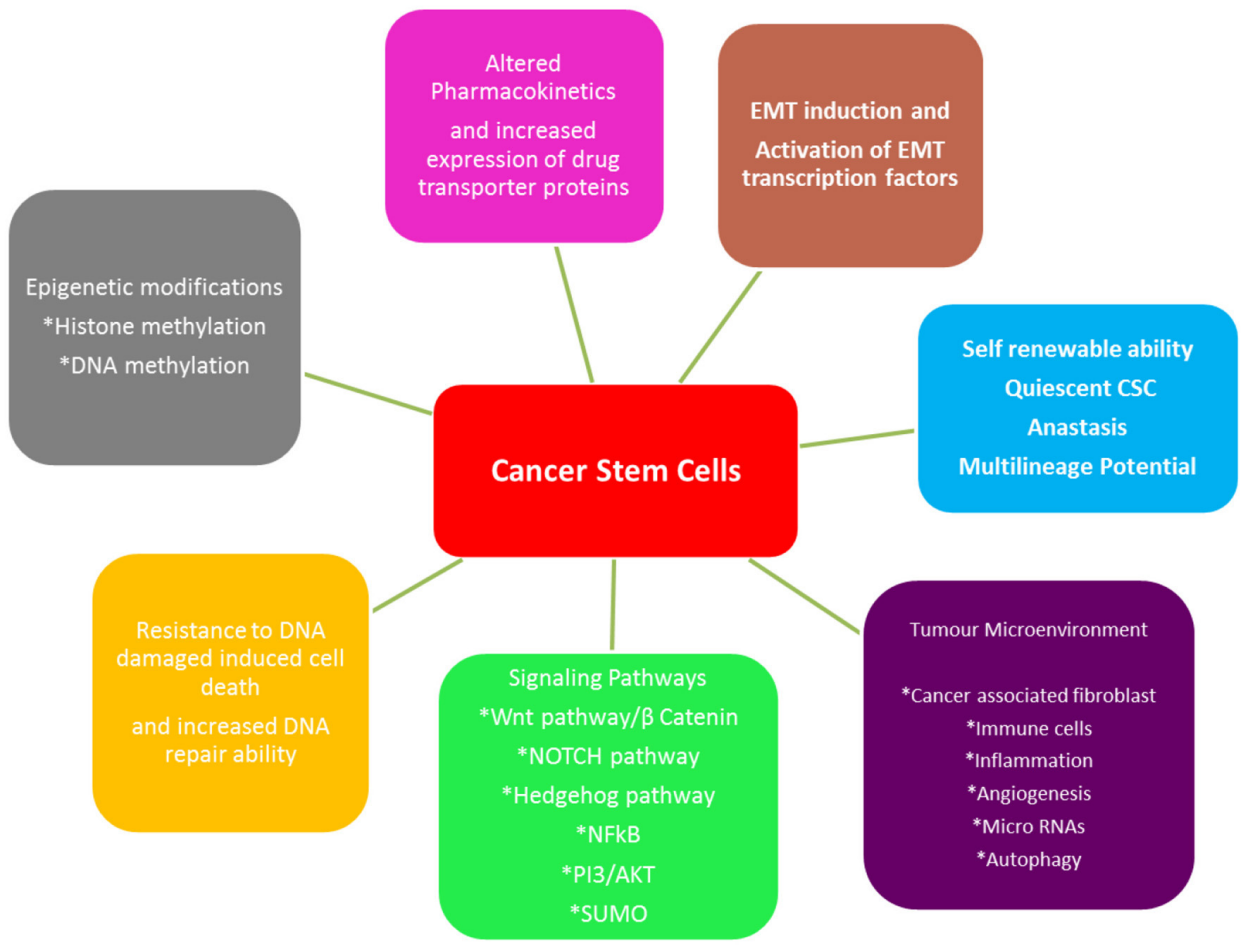
Different mechanisms employed by CSCs for therapeutic resistance (adapted from [184]). CSCs adapt themselves so they can survive the common therapeutic strategies such as chemo- and radiotherapy used to treat oral cancer. These adaptations involve developing mechanisms such as epigenetic modifications, alterations in transport of drug, EMT, stemness, tumor microenvironment, signaling pathways and resistance to apoptosis.

Cancer stem cells (CSCs) are a tumor sub-population serving as progenitors that are capable of self-renewal and production of heterogeneous lineage of cancer cells inside the tumor [178]. The evidence of this sub-population has been reported in several tumors, including oral cancer and play a key role in maintaining tumor population, metastasis and therapeutic resistance [179, 180]. The origin of CSCs has not been clearly defined; in HNSCC, it has been proposed that a chronic inflammation caused by long term use of tobacco, alcohol, mechanic irritation or viral infection, microenviornmental factors in association with genetic predisposition, lead to the accumulation of various genetic lesions and finally to the manifestation of a CSC phenotype. There is a dynamic state of interconversion of non CSCs to CSC under above mentioned circumstances [181]. It is generally accepted that the presence of CSCs make cancers not only to grow but also makes them difficult to treat and enables them to relapse [178]. The mechanism of CSC-mediated therapeutic resistance is still elusive, but they exhibit increased efficiency in DNA repair, overexpression of anti-apoptotic proteins, autophagy, metabolic adaptations alongwith maintaining the lower redox status [182], EMT, altered drug responses; all contributing to the therapeutic resistance and survival of CSCs. Unexpectedly, there are reports suggesting that radiation used in radio-therapy can induce non-CSCs to become CSCs [183]. Different mechanisms through which CSCs play their role in therapeutic resistance are summarized in Figure 5. Common molecular markers reported for the identification of cancer stem cell population are CD44, CD133, CD24, ABCB5, Lin28a, ALDH, phosphorylated STAT3, Nanog, Orail1 and OCT4, C-Met. However, no single CSC-specific marker has been identified yet that can be clinically correlated to the clinical staging of oral cancer or to differentiate them from normal stem cells [185].

### Therapeutic Strategies Targeting CSCs

No distinguishing therapeutic targets for eliminating CSCs have been identified yet. However, different *in-vitro* and *in-vivo* studies have been reported to target critical pathways specific to CSCs such as CD44, c-MET, ALDH1 and Wnt pathways to eradicate CSCs. Radionuclide186Re-cmAb (U36) antibody to target CD44 is in phase I clinical trial in oral cancer patients [178]. Further research is urgently required to identify specific molecular targets and to develop strategies to eliminate CSCs that would curb the cancer growth.

Another survival mechanism by CSC reported recently is the reversal of apoptosis known as anastasis; when the cells revive from “the brim of death or point of no return” after the removal of the apoptosis-inducing agent [186]. The molecular analysis of anastasis has shown upregulated TGF beta, RTK, MAPK and angiogenesis inducing factors. It has been reported that anastasis may be a possible survival pathway of the CSCs to resist the apoptotic death induced by therapeutic agents. Any genetic aberrations causing anastasis in CSCs are most likely to confer additional therapeutic resistance [187].

Recent advances in the field of molecular pathology and genetics have helped better understand the complex and heterogeneous oral cancer molecular profiles. These findings are the basis of recent developments of new therapeutic agents specifically targeting critical molecules or genetic lesions involved in cancer progression and therapeutic resistance. However, there is still no consensus on the specific biomarkers that can be correlated with clinical staging, treatment efficacy and post treatment monitoring of patient recovery [188]. Many critical molecules being studied in other cancers are not being tested in oral cancer; furthermore, number of clinical longitudinal studies on the efficacy of single or combination therapies in oral cancer patients is far fewer. The main focus of the future research should be to explore complex genetic, epigenetic and microenviornmental interactions in oral cancer contributing to treatment failure especially the CSCs that are now accepted as the root cause of cancer relapse and therapeutic resistance.

## Conclusion

Oral cancer is a heterogeneous, aggressive and complex entity. Main treatment options in practice are surgery, chemo-, radio-, and immunotherapy alone or in combination. Each treatment modality has its own limitations. Surgical interventions lead to significant loss of oral functions and that is followed by multiple corrective surgeries which lead to substantial disfigurement with a long journey to rehabilitation. This invariably leads not only to loss of self-esteem but also considerable pain and suffering to loved ones. Other therapies such as chemotherapy, radiotherapy, immunotherapy etc. have limitations in-terms of cytotoxicity, tolerance, non-specificity, resistance and loco-regional relapse. Modern research has unveiled a new mutational landscape of oral cancer and factors contributing to the therapeutic resistance. In light of these new findings, combination therapies should be utilized rather than single therapy based on the individual's specific molecular signature of cancer to overcome treatment resistance. In this way treatment can be better tailored for patients and resistance can be minimized. In this review we have discussed major factors contributing to therapy resistance and treatment failure in oral cancer such as genetic factors, signaling pathways, CSC and DNA damage repair system hyperactivation. More research is needed to explore novel genetic alterations and complex extracellular microenvironmental factors such as hypoxia, exosomes, immune cells, angiogenic factors (not mentioned in this review) etc. responsible for therapeutic resistance and relapse of oral cancer as there are still many unexplored avenues. In this way, more effective personalized targeted therapies can be researched and made available to patients.

## Author Contributions

SU, AW, and M-TT: conceived the idea and wrote the manuscript. AW, M-TT, and AJ: edited and revised the manuscript. All authors contributed to the article and approved the submitted version.

## Conflict of Interest

The authors declare that the research was conducted in the absence of any commercial or financial relationships that could be construed as a potential conflict of interest.
